# Bilateral external iliac artery pseudoaneurysms causing urinary obstruction and acute renal failure

**DOI:** 10.1186/s42155-022-00302-5

**Published:** 2022-05-25

**Authors:** Marcelo Puppo Bigarella, Roberto Iglesias Lopes, Guilherme Gentile, Carolina Brito Faustino, Lais da Cunha Gamba, Guilherme Baumgardt Barbosa Lima, Henry Augusto Hoffman Melo, Grace Carvajal Mulatti

**Affiliations:** 1 Division of Urology, Department of Surgery, University of São Paulo School of Medicine, Rua Helena 218 4 floor 410 – Sartor Medicina, São Paulo, 04552-050 Brazil; 2 Division of Vascular Surgery, Department of Surgery, University of São Paulo School of Medicine, São Paulo, Brazil

**Keywords:** Ruptured pseudoaneurysm, External iliac artery, Ureteral obstruction, Bilateral ureterohydronephrosis, Endovascular repair

## Abstract

**Background:**

We report an exceedingly rare case of bilateral external iliac artery pseudoaneurysms causing urinary obstruction and acute renal failure.

**Case presentation:**

A young man presented with acute severe bilateral testicular pain radiating to the back. Clinical and radiological workup showed bilateral external iliac artery pseudoaneurysms, which caused bilateral ureterohydronephrosis due to urinary obstruction with subsequent renal failure. Management included immediate bilateral external iliac artery endovascular repair and bilateral ureterolysis using a retroperitoneal approach, with resolution of the obstruction and successful endovascular treatment of both pseudoaneurysms. The only identifiable risk factor for cardiovascular disease was cocaine addiction.

**Conclusions:**

This case highlights an unusual and severe clinical presentation of bilateral EIA pseudoaneurysms causing bilateral ureterohydronephrosis and subsequent renal failure. Awareness of this condition may help avoid misdiagnosis and delayed management, which is of utmost importance for a favorable outcome.

## Background

Pseudoaneurysms are usually caused by trauma, infection, vasculitis, atherosclerosis, or iatrogenic complications (Sueyoshi et al. 2005; Lazarides et al. 1991), with most cases remaining clinically silent. However, a few may become symptomatic, and patients might present with limb ischemia, leg edema, and subacute or acute hemorrhage. An unusual presentation of bilateral external iliac artery (EIA) pseudoaneurysms is reported here.

## Case presentation

A 29-year-old man presented to the emergency department with acute severe bilateral testicular pain radiating to the back. The pain initiated during the night, awakening the patient and prompting medical attention. He described the pain as sharp and continuous, radiating to his lower back bilaterally with no worsening or alleviating factors. The patient had no history of trauma, underlying medical conditions, regular medications, or previous surgical procedures. Abnormal urinary symptoms were also ruled out.

On admission, he was hypotensive (100/60 mmHg) with normal heart rate and normal neurological status. Physical examination revealed mild dehydration, pale mucous membranes, and cool extremities. Peripheral pulse was symmetric in the lower and upper limbs. No heart murmurs, signs of pulmonary congestion or pleural effusion were observed. On abdominal palpation, a lower abdominal mass was noted with mild discomfort. No signs of acute peritonitis were present. Testicular examination was unremarkable.

Anemia (hemoglobin level of 7.6 g/dL and hematocrit of 24%) and acute renal failure (creatinine of 2.8 g/dL, normal blood urea with an estimated glomerular filtration rate of 29 mL/min/1.73 m^2^) were observed. Testicular tumor markers were negative. Abdominal ultrasound revealed a retroperitoneal mass causing bilateral ureterohydronephrosis. All imaging studies were normal.

**Fig. 1:**
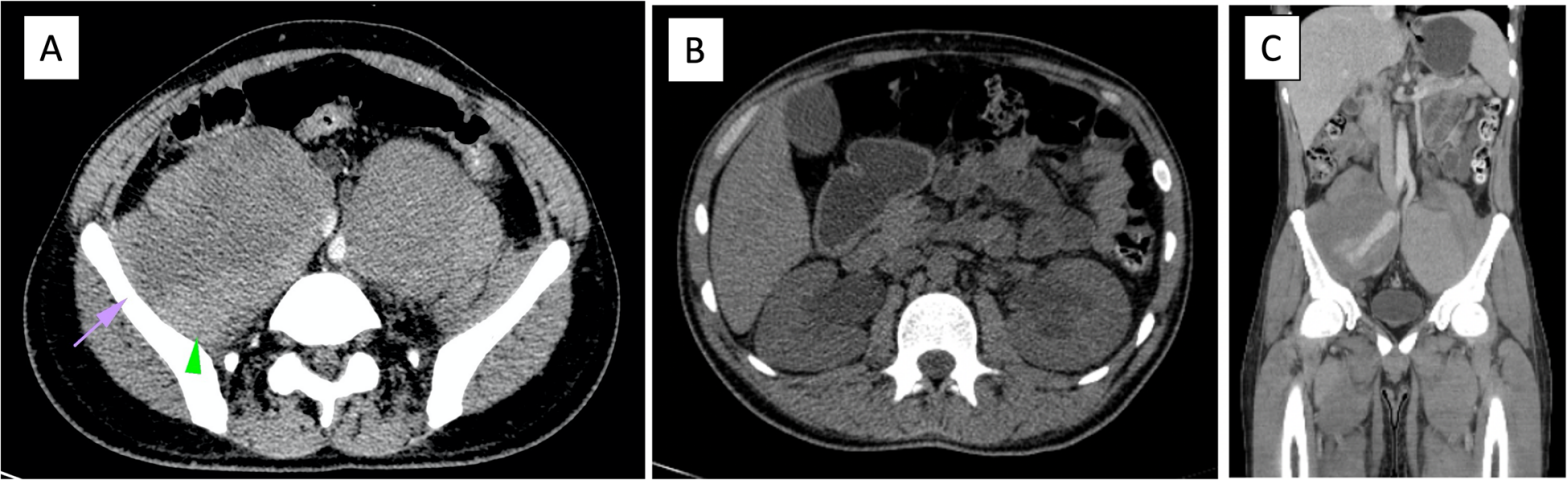
Contrast-enhanced CT scan of the abdomen and pelvis. Axial view showing bilateral large-diameter pseudoaneurysms of the external iliac arteries; **A** the arrowhead shows a pelvic hematoma with increased attenuation and the arrow shows a draping effect on the posterior external iliac wall. **B** axial view, demonstrating bilateral ureterohydronephrosis; **C** Coronal view, in which leakage of contrast is seen in the right side

Even in the setting of acute renal failure, contrast-enhanced multiaxial tomography of the abdomen and pelvis was deemed necessary to establish a definitive diagnosis. It revealed bilateral EIA pseudoaneurysms (Fig. 1A) causing moderate bilateral ureterohydronephrosis (Fig. 1B) with clear signs of contrast leakage from the right EIA pseudoaneurysm into the retroperitoneum, reflecting evidence of an acutely ruptured pseudoaneurysm associated with an ipsilateral retroperitoneal hematoma (Fig. 1C). These findings supported acute anemia and renal failure.

**Fig. 2:**
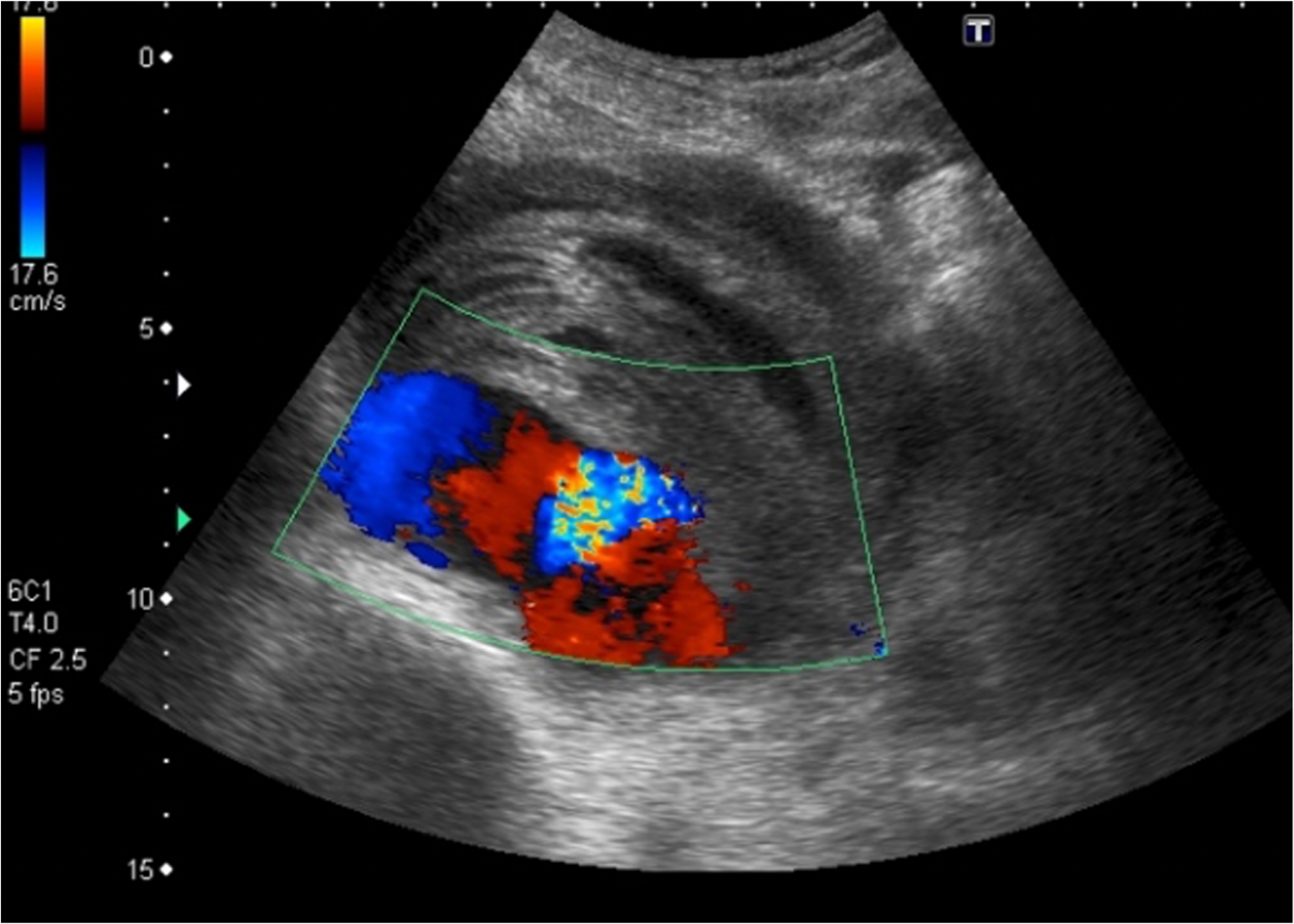
Doppler ultrasound of the right external iliac artery showing a 10.6 × 9.4 cm pseudoaneurysm with turbulent flow to the posterior iliopsoas region, compatible with rupture

The patient was blood typed, and saline expansion was performed. A left jugular vein catheter was placed in case of acute deterioration. The on-call vascular surgeon performed a bedside Doppler ultrasound of the iliac arteries revealing a turbulent flow with swirling motion pattern, also known as the “yin-yang sign,” in the right EIA, thus confirming the finding of a ruptured pseudoaneurysm of the right EIA (Fig. 2).

**Fig. 3:**
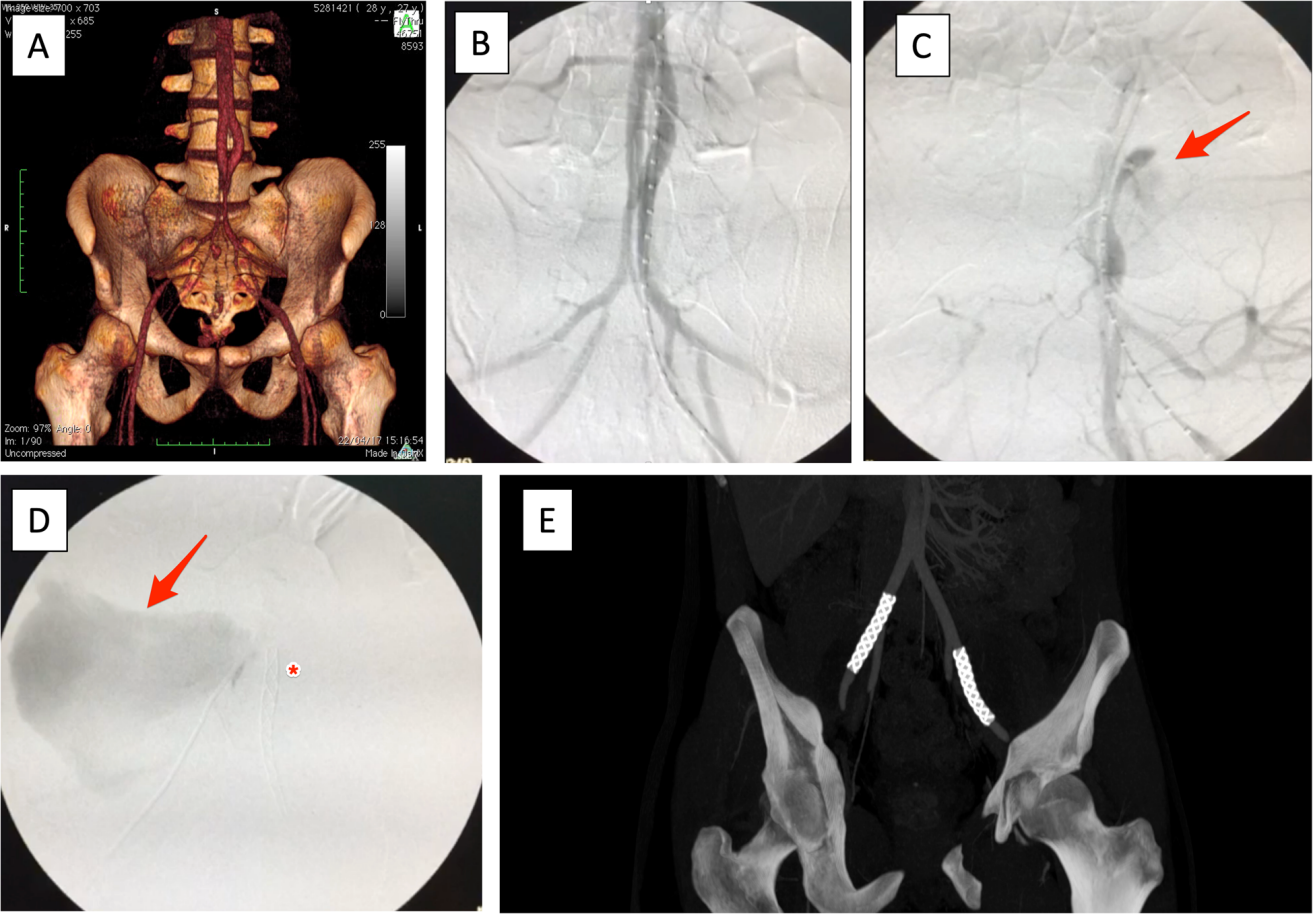
**A** Preoperative 3D vessel reconstruction; **B** Intraoperative arteriogram of the iliac arteries; **C** Selective arteriogram of the left external iliac artery pseudoaneurysm, oblique view, arrow points the leakage; **D** Selective arteriogram of the right external iliac artery pseudoaneurysm showing contrast leakage (arrow) – with left endoprosthesis in place (*); **E** Control CT with no evidence of contrast leakage, a week later

Due to the risk of ongoing bleeding, the patient underwent emergency endovascular repair of the bilateral EIA pseudoaneurysms (Fig. 3A, B, and C), ruptured on the right side (Fig. 3D), with the placement of bilateral 8 mm (in diameter) × 50 mm (in length) WALLGRAFT® endoprostheses (Boston Scientific, Marlborough, MA, USA). After angiographic confirmation of successful treatment, with no further bleeding or leakage (Fig. 3E and F), open inguinotomy was performed for ureterolysis and drainage of retroperitoneal hematoma.

Immediate post-decompression diuresis was observed, with substantial urine output (12 L) in the first 24 hours after the procedure. Creatinine peaked at 3.2 g/dL on postoperative day 1, and his renal function normalized after 1 week of follow-up. During this period, the patient was closely monitored for his hemodynamic condition and renal function recovery to prevent dehydration and electrolyte imbalance.

Hematoma content and connective tissue removed from the perianeurysmal area were analyzed histopathologically and tested for bacteria and fungi. Serology for community-acquired infectious diseases, inflammatory laboratory tests and other specific rheumatological tests (antinuclear antibodies for systemic lupus erythematosus and other autoimmune tests) were performed, and the patient was evaluated by a multidisciplinary panel of experts in the fields of vascular surgery, internal medicine, infectious disease, rheumatology, and urology. Laboratory results were within the normal range, and cultures were negative. The only identifiable risk factor for cardiovascular disease was cocaine addiction (for the past 2 years).

## Conclusion

Bilateral ureteral obstruction and acute renal failure due to bilateral iliac artery conditions is a very rare entity, with only a few reported cases (Wu and Ma 2019; Oliveira et al. 2018). This observation might be explained by the reported prevalence of this condition. The occurrence of iliac artery aneurysms associated with abdominal aortic aneurysms is relatively common, accounting for 10% to 20% of all aortic aneurysms (Uberoi et al. 2011). Isolated iliac artery aneurysms, however, are rare (0.4% to 1.9% of all intra-abdominal aneurysms) (Richardson and Greenfield 1988), with EIA aneurysms being even rarer (Uberoi et al. 2011) and mostly associated with a previous history of trauma or infection (Hussain and Aziz 2019; Krupski et al. 1998).

To our knowledge, this is the first reported case of ruptured EIA pseudoaneurysms causing bilateral ureterohydronephrosis with subsequent renal failure. An unusual clinical presentation as acute severe bilateral testicular pain radiating to the back, compensated hypovolemic shock, acute anemia and acute kidney injury were the most remarkable early findings in this particular case.

Common underlying causes of pseudoaneurysm were excluded, and the only identifiable risk factor for cardiovascular disease was cocaine addiction. This clinical presentation is worrisome, and we believe this case merits report because misdiagnosis might result in a significant delay in the treatment of a critical emergency condition. Hypovolemic shock may occur in case of rupture; therefore, prompt management is crucial with either an open or an endovascular approach. Endovascular intervention using a covered stent has the advantages of being less invasive and achieving faster bleeding control in most cases (Adovasio et al. 2003).

In the present case, relief of urinary obstruction was also necessary. Hematoma drainage using an open retroperitoneal approach and bilateral ureterolysis were required to resolve the ureteral entrapment caused by the inflammatory pseudoaneurysms. An open repair was also of interest in order to obtain material for histopathological analysis.

This case highlights an unusual and severe clinical presentation of bilateral EIA pseudoaneurysms causing bilateral ureterohydronephrosis and subsequent renal failure. Radiological workup showed a ruptured pseudoaneurysm on the right side although the patient was still on the compensated phase of hypovolemic shock. Awareness of this condition may help avoid misdiagnosis and delayed management, which is of utmost importance for a favorable outcome.

## Data Availability

Data sharing is not applicable to this article as no datasets were generated or analyzed during the current study.
